# The Association Between Perceived Ability to Use Artificial Intelligence, Self-Reported Nursing Productivity, and Self-Reported Practice Readiness Among Nurses in Saudi Arabia: A Multicentre Cross-Sectional Study

**DOI:** 10.3390/healthcare14183065

**Published:** 2026-09-17

**Authors:** Ayman Mohamed El-Ashry, Eidah Farhan Ayed Alanazi, Mahmoud Abdelwahab Khedr, Ahmed Abdellah Othman, Samah Mohammed Baaqil, Norah Hadi Alwadei, Fatma A. Rajhi, Abdullah Suwailem Alsharari, Hamdah Farhan Ayed Alanazi, Wejdan Abdulrahman Almutairi, Shamsh Farhan Ayed Alanazi, Shrouq Farhan Ayed Alanazi, Badr Ayed Alenazy, Maha Farhan Ayed Alanazi, Hanadi Saad Bin Talib, Khloud Abdulhadi Alshehri, Zahra Abdulsalam Alsinan, Nora Ghalib AlOtaibi

**Affiliations:** 1 Psychiatric and Mental Health Nursing, Faculty of Nursing, Alexandria University, Alexandria 21500, Egypt; mkhader@uhb.edu.sa; 2 Department of Nursing, College of Applied Medical Sciences, Jouf University, Al-Qurayyat 77452, Saudi Arabia; 3 Riyadh Second Health Cluster, Ministry of Health, Riyadh 11525, Saudi Arabia; eidahfa@moh.gov.sa (E.F.A.A.); hamdahfa@moh.gov.sa (H.F.A.A.); 4 Nursing Administration, Faculty of Nursing, Sohag University, Sohag 82511, Egypt; ahmed_abda@nursing.sohag.edu.eg; 5 Nursing Administration, Al-Aziziyah Children’s Hospital, Jeddah Health Cluster, Ministry of Health, Jeddah 23342, Saudi Arabia; sbaaqil@moh.gov.sa; 6 Eastern Health Cluster, Ministry of Health, Dammam 32253, Saudi Arabia; nhalwadei@moh.gov.sa (N.H.A.); zaalsinan@moh.gov.sa (Z.A.A.); 7 Jazan Health Cluster, Ministry of Health, Jazan 82723, Saudi Arabia; al-raheel27@hotmail.com; 8 Aljouf Health Cluster, Ministry of Health, Sakaka 72341, Saudi Arabia; aalshrari@moh.gov.sa; 9 Dr. Sulaiman Al Habib Hospital, Ministry of Health, Buraydah 52381, Saudi Arabia; wejdan_7n@hotmail.com; 10 College of Nursing, Riyadh Elm University, Riyadh 12734, Saudi Arabia; shmos8h@gmail.com; 11 King Khalid University Hospital, Ministry of Health, Riyadh 11472, Saudi Arabia; shrouq@ksu.edu.sa; 12 Northern Borders Health Cluster, Ministry of Health, Arar 73211, Saudi Arabia; balenazy@moh.gov.sa; 13 Nursing Department, Branch-Riyadh Region, Ministry of Health, Riyadh 11176, Saudi Arabia; malanazi51@moh.gov.sa; 14 Nursing Department, King Salman bin Abdulaziz Medical City, Ministry of Health, Al Madinah 42319, Saudi Arabia; hbintalib@moh.gov.sa; 15 Council of the Doctor of Philosophy in Nursing Program, King Saud University, Riyadh 12372, Saudi Arabia; khloudalshehri17@gmail.com; 16 Department of Community Nursing, College of Nursing, Imam Abdulrahman Bin Faisal University, Dammam 31441, Saudi Arabia; ngalotaibi@iau.edu.sa

**Keywords:** artificial intelligence, nursing productivity, practice readiness, health workforce, technology adoption, cross-sectional studies, Saudi Arabia

## Abstract

**Highlights:**

**What are the main findings?**

- Higher perceived ability to use AI was positively correlated with both self-reported nursing productivity and self-reported practice readiness in a convenience sample of 250 nurses working in Saudi Arabia.
- Perceived ability to use AI remained positively associated with self-reported nursing productivity and self-reported practice readiness after covariate adjustment. Because the design is cross-sectional and all three constructs were self-reported, these findings do not establish causal effects.

**What are the implications of the main findings?**

- The findings are consistent with, but do not establish, a case for usable, workflow-aligned AI tools supported by structured nurse training and technical support.
- AI literacy for nurses should cover critical appraisal of AI output, verification against reliable sources, privacy, accountability, bias, and the limits of general-purpose generative tools, rather than tool operation alone.

**Abstract:**

Background/Objectives: Artificial intelligence (AI) tools are increasingly available to nurses, but it is unclear how nurses’ perceived ability to use them relates to self-reported work outcomes in Saudi Arabia. This study examined the associations of perceived ability to use AI with self-reported nursing productivity and self-reported practice readiness. Methods: A multicentre cross-sectional survey was conducted between January and March 2025 among a convenience sample of 250 nurses working in general hospitals in five Saudi cities or regions, and it was reported in accordance with the STROBE statement. The questionnaire comprised sociodemographic, professional and AI-use items, the eight-item “Perceived ability to use AI” subscale, the six-item Nursing Productivity Scale and the Nursing Practice Readiness Scale (NPRS). Analyses used Spearman correlations with bootstrap confidence intervals, Mann–Whitney and Kruskal–Wallis tests with Benjamini–Hochberg control of the false discovery rate, and multivariable linear models with HC3 heteroscedasticity-consistent standard errors, reported as unstandardized coefficients with 95% confidence intervals. Results: Mean scores were 27.95 (SD, 7.94) for perceived ability to use AI, 21.50 (SD, 5.42) for productivity and 109.92 (SD, 23.77) for practice readiness. Perceived ability to use AI was correlated with productivity (r_s = 0.638, 95% CI 0.529 to 0.727) and with practice readiness (r_s = 0.479, 95% CI 0.363 to 0.580), and productivity was correlated with readiness (r_s = 0.642, 95% CI 0.548 to 0.721). In the adjusted model with HC3 standard errors, each additional point of perceived ability to use AI was associated with 0.38 points of productivity (95% CI 0.28 to 0.49) and 0.80 points of readiness (95% CI 0.45 to 1.14). Conclusions: Nurses who rated their ability to use AI more highly also rated their own productivity and practice readiness more highly. The cross-sectional design, convenience sampling, common-method variance and the absence of any objective performance or patient outcome mean that these findings are associations among self-reports.

## 1. Introduction

Artificial intelligence (AI) tools are becoming part of the everyday working environment of nurses, and interest in what that means for care delivery, workload and professional development has grown quickly [1,2]. Nurses are the largest professional group in health systems, with roughly 30 million nurses worldwide and 184,565 registered nurses in Saudi Arabia [3,4], so how they engage with these tools matters at scale. Saudi health-sector reform under Vision 2030 places digital transformation, e-health and data use at the centre of service modernisation [5].

Two terms need to be separated at the outset. Usability is a property of a specific system used by defined users for a defined task, and it is assessed through that interaction rather than through a general opinion about a class of technology [6,7]. What the present study measured is different and narrower: nurses’ perceived ability to use AI tools in their own nursing work. Nursing productivity is understood here as nurses’ own appraisal of how timely, efficient and complete their care delivery is, which typically reflects administrative load, workload distribution and the share of the shift spent on direct care [1]. Practice readiness refers to a nurse’s perceived preparedness to carry out professional nursing duties safely and independently, including clinical judgement, professional attitude, patient-centredness, self-regulation and collaboration [8,9]. Readiness in this sense is not specific to AI; it is a general professional self-appraisal [10]. Reviews of AI in nursing report that where AI functions are embedded in the electronic health record and in existing documentation and decision-making workflows, nurses describe smoother workflows and a lower documentation burden, whereas standalone or poorly fitted tools add work [11,12].

Vision 2030 and the Health Sector Transformation Program identify digital transformation, e-health and AI-supported innovation as routes to better access, quality and efficiency of care. National programmes promote telemedicine and smart documentation, and these in turn assume a workforce that is comfortable with such technologies [5,13]. What such programmes describe is the institutional deployment of clinical systems. That is not what the participants in this study reported using, and the distinction is maintained throughout the manuscript.

Saudi and regional studies report that nurses and nursing students generally hold favourable views of AI, tend to report only moderate technological readiness, and ask for more training and better system design [9,14]. Qualitative work suggests that nurses expect AI to reduce workload where the interface is convenient and does not add documentation or alert fatigue, and to add work where it is not [2,15]. Almost all this evidence concerns perceptions and intentions rather than measured use or measured outcomes, which is the context in which the present study should be read.

The gap this study addresses is therefore specific. Saudi nurses now have wide access to general-purpose generative AI tools, yet almost all of the existing Saudi evidence concerns attitudes towards, or intention to adopt, AI among students, and very little of it examines whether nurses who feel more able to use these tools also report different work outcomes [1,14,16]. To our knowledge, no Saudi study has examined perceived ability to use AI alongside self-reported productivity and self-reported practice readiness in the same sample of practising nurses. The aim of this study was to describe those associations. The design is cross-sectional and every variable is a self-report, so the study cannot establish direction or causation, and it was not designed to evaluate any AI system.

### 1.1. Theoretical Background

Task technology fit (TTF) is used in this manuscript as an interpretive lens only, not as a tested model. TTF holds that a technology improves individual performance to the extent that its functions match the requirements of the task the user must perform [17,18]. The present study measured none of the three TTF constructs: it did not characterise the technology, did not measure task characteristics and did not measure the fit between them. TTF is therefore used here to explain why a nurse’s own sense of being able to work with these tools might accompany a more favourable appraisal of their own work, and no result reported below should be read as confirming or testing TTF.

Within that limited framing, the study addressed two research questions and their corresponding hypotheses. H1: perceived ability to use AI is positively correlated with self-reported nursing productivity. H2: perceived ability to use AI is positively correlated with self-reported practice readiness. Additional multivariable analyses examined whether these associations persisted after adjustment for the covariates described in Methods.

### 1.2. Aim, Research Questions and Hypotheses

The aim of this study was to examine the associations of perceived ability to use AI with self-reported nursing productivity and self-reported practice readiness among nurses working in Saudi Arabia.

The research questions were:

RQ1. Is perceived ability to use AI correlated with self-reported nursing productivity among nurses working in Saudi Arabia?

RQ2. Is perceived ability to use AI correlated with self-reported practice readiness in the same sample?

## 2. Materials and Methods

### 2.1. Study Design, Setting and Data Collection

This was a multicent cross-sectional survey, reported in accordance with the Strengthening the Reporting of Observational Studies in Epidemiology (STROBE) statement [19]. Data were collected between 1 January and 31 March 2025 from nurses working in government general hospitals in five Saudi cities or regions: Riyadh, Jouf, Jeddah, Al Ahsa and Arar. Data were collected from five general hospitals with bed capacities ranging from 150 to 200 beds and reported nurse-to-patient ratios of approximately 1:5 on general wards and 1:1 in critical care. Sites were not sampled at random: they were the facilities at which members of the research team held appointments or had administrative permission to recruit, which makes the arrangement a convenience one at the site level as well as at the participant level.

Data were collected by trained members of the study team using a structured, self-administered paper questionnaire. All participants completed the same self-administered instrument; the earlier description of the sociodemographic sheet as “researcher-administered” was an error and has been corrected. A data collector was present to clarify instructions but did not read items aloud or record answers. Nurses were approached face to face inside the facility, between shifts, and consenting participants completed the questionnaire in a quiet staff room or education room. Recruitment followed the institutional condition that staff be invited by direct approach or administrative circulation and that researchers not obtain personal contact details. Of the 265 nurses invited, 12 declined and 3 were excluded because their questionnaires were substantially incomplete, giving *n* = 250 analysed and a response rate of 96%. There were no missing values in the analysed dataset, because questionnaires that were incomplete on any scale item were excluded at data entry rather than imputed.

### 2.2. Sample Size, Sampling and Participants

An a priori sample size estimate was obtained in G*Power version 3.1.9.7 for linear multiple regression (fixed model, R^2^ deviation from zero, F test). The prespecified adjusted model contains eleven conceptual variables, ten of which are categorical; after dummy coding these correspond to 30 predictor degrees of freedom, so 30 predictors were entered. With α = 0.05, power = 0.80 and f^2^ = 0.15, the minimum required sample was 187. The achieved sample of 250 exceeds this, but 30 predictor degrees of freedom in 250 observations remains a demanding specification, and several categories are small. For that reason, a prespecified reduced model, in which sparse categories are combined on clinical grounds, is reported alongside the full model as the primary sensitivity analysis (Section 2.5). Participants were recruited by convenience sampling. Nurses were eligible if they were employed at a participating hospital during the data-collection period, were available during the recruitment visit and gave informed consent. Non-nursing staff, nurses who declined and substantially incomplete questionnaires were excluded. Because recruitment was by convenience and took place at sites already engaged with the research team, nurses with an existing interest in AI are likely to be over-represented; this is the most plausible explanation for the very high proportions reporting previous AI training (88.0%) and endorsing an AI course (96.0%), and these proportions are treated as a limitation rather than as population estimates.

### 2.3. Measures

#### 2.3.1. Sociodemographic, Professional and AI-Use Questionnaire

This section of the self-administered questionnaire recorded gender (male, female); city or region (Arar, Al Ahsa, Jeddah, Jouf, Riyadh); age, which was collected as three ordered categories (<25, 25–35, ≥36 years) rather than as a continuous variable, so that no mean age can be reported; highest educational qualification (diploma, bachelor, master, doctorate); job title (assistant nurse, registered nurse, charge nurse, head nurse, nursing education, quality nurse, infection control nurse); nursing specialty (acute, emergency, intensive care, primary health care, surgical, midwifery, oncology, general); and years of experience, collected as a binary variable (<5, ≥5 years). Three AI-use items were included: the AI application used most often (single choice: ChatGPT 4.1, Gemini 2.5, DeepSeek-R1 or others, with a free-text field that was not coded further); previous attendance at any training course on AI use in nursing (yes/no); and whether the respondent considered it necessary to incorporate an AI course into nursing practice (yes/no). These three items were all that was captured about AI use. The questionnaire did not record the purpose of use, the frequency or duration of use, whether use was clinical or non-clinical, whether it was individually initiated or institutionally sanctioned, whether the tool was integrated into the electronic health record or any other clinical system, whether patient data were entered, whether human supervision was in place, or the access modality (free or paid, institutional or personal). It also did not offer a non-user response option, so participants who did not use AI at all could not be identified. Nor did it record the content, duration, provider or recency of the training reported in the second item. This is a fundamental limitation of the exposure measurement, and it carries through to the interpretation of every result below.

#### 2.3.2. Perceived Ability to Use AI

This construct was measured with the eight-item “utilization of AI in nursing jobs” subscale of the Arabic instrument validated by Alenezi et al. (2024) [12], which was itself adapted from the Medical Artificial Intelligence Readiness Scale for Medical Students (MAIRS-MS) [20]. Only this subscale was administered, for two reasons: it is the only part of the parent instrument that has been adapted and psychometrically evaluated in Arabic among nurses [12], and the remaining MAIRS-MS domains address medical-student curricular preparedness that does not transfer to practising nurses. Administering a subscale rather than the full instrument means that the broader MAIRS-MS construct was not captured, and this is acknowledged as a limitation in Section 4.2. All eight items, in Arabic and in English translation, are provided in Supplementary Table S1 so that readers can judge what was measured. The items ask respondents to rate their own ability to use AI applications in their nursing work; they do not ask about the effectiveness, efficiency, learnability, error tolerance or workflow integration of any system. The label “AI usability” used in the previous version of this manuscript was therefore incorrect and has been replaced throughout with “perceived ability to use AI”, including in the title. Responses are given on a five-point Likert scale from 1 (strongly disagree) to 5 (strongly agree), giving a total score of 8 to 40, with higher scores indicating greater perceived ability. In the source study the item loadings ranged from 0.791 to 0.858, composite reliability was 0.938, average variance extracted was 0.684 and Cronbach’s alpha was 0.923 [12]. In the present sample Cronbach’s alpha was 0.952, McDonald’s omega 0.952, composite reliability 0.952 and average variance extracted 0.714, with one-factor loadings from 0.82 to 0.89 and a standardised root mean square residual of 0.029 for the congeneric one-factor solution.

#### 2.3.3. Nursing Productivity Scale

Self-reported productivity was measured with the six-item scale developed by Lutwama (2011) [21] and translated into Arabic and validated by Alenezi et al. (2024) [12]. Items are rated from 1 (strongly disagree) to 5 (strongly agree), giving a total score of 6 to 30, with higher scores indicating higher self-appraised productivity. In the source study item loadings ranged from 0.544 to 0.847, composite reliability was 0.835, average variance extracted was 0.566 and Cronbach’s alpha was 0.741 [12]. In the present sample Cronbach’s alpha was 0.869, McDonald’s omega 0.869, composite reliability 0.869 and average variance extracted 0.529, with one-factor loadings from 0.58 to 0.82. This is a perceptual measure. No objective productivity indicator (documentation time, tasks completed, workload, time in direct care, errors or rework) was collected, and the scale should not be read as a measure of actual output.

#### 2.3.4. Nursing Practice Readiness Scale

Self-reported practice readiness was assessed using the Arabic Nursing Practice Readiness Scale (NPRS) [22,23]. The version administered and analysed here contains 34 items, each rated from 1 (strongly disagree) to 4 (strongly agree). The original developers score the NPRS as overall and subscale means across five factors (clinical judgement and nursing performance, professional attitude, patient-centredness, self-regulation, and collaborative interpersonal relationships). A total sum score is used here because the total is a linear transformation of the overall mean and therefore gives identical correlations and identical inference, and because the five-factor structure did not reproduce in this sample. Both the instrument and the Arabic validation were developed for nursing students and newly graduated nurses in transition to practice [22,23], whereas 61.2% of the present sample had five or more years of experience. Applying the scale to experienced nurses’ changes what the score plausibly means, and this was examined empirically rather than assumed. In the present sample Cronbach’s alpha was 0.983, McDonald’s omega 0.983, composite reliability 0.983 and average variance extracted 0.636. The Kaiser Meyer Olkin measure was 0.956. The first unrotated component accounted for 65.0% of item variance, with an eigenvalue of 22.09 against 1.31 for the second, and parallel analysis against 200 random datasets retained one factor rather than five. The mean inter-item correlation was 0.637 (range 0.38 to 0.83). Taken together, these results indicate that in this sample of practising nurses the NPRS behaves as a single, highly redundant self-appraisal dimension rather than reproducing the five-factor structure reported in students. The total score is therefore interpreted here as a general positive self-appraisal of professional practice, and it is described throughout as “self-reported practice readiness” rather than as readiness to enter practice. An alpha of 0.983 combined with a mean inter-item correlation of 0.637 is evidence of item redundancy, not of validity.

### 2.4. Ethical Considerations

Ethical approval for this multicentre cross-sectional study was obtained from the Riyadh Second Health Cluster Institutional Review Board on 20 August 2024 (IRB Log No. 24-467E), with approval conditions specifying the requirements for amendments, administrative and site permissions, document retention, and periodic reporting. Participation was fully voluntary. Eligible nurses were informed about the study aim, the procedures and their rights before participation, and informed consent was obtained before data collection. Participants were informed that they could decline participation or withdraw at any time without any consequences for their work status or evaluations. Confidentiality and privacy protections were applied throughout. Questionnaires were collected without personal identifiers where possible, and all study materials were stored securely, with access limited to the research team. Data were used strictly for research purposes and reported only in aggregate form to prevent the identification of individuals or specific work units. Recruitment and approach procedures complied with institutional requirements, including the condition that staff invitations occur through direct approach at the facility or administrative circulation and that researchers not obtain staff personal contact details unless the staff member provided written permission.

### 2.5. Statistical Analysis

Analyses were run in IBM SPSS Statistics 27.0, with the robust-variance and resampling steps reproduced in R 4.3. All analyses reported below were completed before resubmission; nothing is deferred. Categorical variables are summarised as frequencies and percentages, and the three scale scores as mean, standard deviation, median, interquartile range, minimum and maximum, because all three departed from normality (Shapiro–Wilk *p* < 0.001 for each; skewness −0.74, −0.85 and −1.40, respectively).

Reliability and internal structure. Cronbach’s alpha, McDonald’s omega, composite reliability, average variance extracted, the mean inter-item correlation and a congeneric one-factor maximum-likelihood solution (with its standardised root mean square residual) were computed for each of the three scales. For the NPRS, which was developed in students and applied here to practising nurses, the internal structure was examined further through the eigenvalues of the item correlation matrix, the Kaiser–Meyer–Olkin measure and parallel analysis against 200 simulated random datasets of the same dimensions.

Bivariate analysis. Correlations among the three scale scores are Spearman rank coefficients with percentile bootstrap 95% confidence intervals from 5000 resamples. Group comparisons in Table 1 use the Mann–Whitney U test for two-group comparisons and the Kruskal–Wallis H test for multi-group comparisons, replacing the t tests and ANOVAs of the previous version, because the outcome distributions are skewed and several groups are small and unequal in size. Effect sizes are the rank-biserial correlation for two groups and epsilon-squared for more than two. Table 1 contains 30 tests, all of them exploratory; *p* values are therefore accompanied by Benjamini–Hochberg false-discovery-rate q values computed across the full family of 30, and only q < 0.05 is treated as noteworthy. Where an overall Kruskal–Wallis test was significant, Dunn post hoc comparisons with Benjamini–Hochberg adjustment were performed. Bivariate correlations were used to test H1 and H2, and multivariable models examined the corresponding associations after covariate adjustment. All comparisons in Table 1 were exploratory.

Adjusted models. Two multivariable linear models were fitted, one for total productivity and one for total practice readiness. The exposure, perceived ability to use AI, was entered as a continuous covariate and the ten prespecified categorical covariates as factors, main effects only. Because model diagnostics showed heteroscedasticity (Breusch–Pagan LM = 113.8, df = 30, *p* < 0.001 for productivity; LM = 161.5, df = 30, *p* < 0.001 for readiness), all inference is based on HC3 heteroscedasticity-consistent standard errors rather than on conventional ordinary-least-squares tests. Type III sums of squares were used for the omnibus tests because the design is non-orthogonal and cell sizes are markedly unequal, so that each term is tested after every other term; the omnibus F and partial eta-squared values are retained in Supplementary Table S2 only as a summary of overall contribution. The estimates that carry the interpretation are the unstandardized regression coefficients with HC3 95% confidence intervals reported in Table 2, because Type III tests and partial eta-squared values indicate that a term contributes but say nothing about the direction or magnitude of its association.

Diagnostics and sensitivity analyses. Multicollinearity was assessed by variance inflation factors, influence by leverage and Cook’s distance, and linearity and constant variance by inspection of residual plots. Four prespecified sensitivity analyses were run: (i) a reduced model in which sparse categories were combined on clinical grounds before analysis (education as diploma, bachelor, postgraduate; job title as bedside or staff, supervisory, and educator, quality or infection control; specialty as critical or acute, surgical, oncology or midwifery, and primary health care or general; most-used programme as ChatGPT 4.1, Gemini 2.5, other or unspecified); (ii) refitting after excluding observations with Cook’s distance above 4/n; (iii) refitting without the two extremely imbalanced AI-attitude variables, since the reference groups contain only 30 and 10 participants; and (iv) cluster-robust standard errors with the city or region as the clustering unit. The fourth analysis is an approximation only. Hospital identifiers were not retained in the analysis file, so within-hospital dependence cannot be modelled directly, and with five clusters the cluster-robust-variance estimator is itself unreliable; both points are stated as limitations rather than presented as solutions.

Moderation. Product terms between the mean-centred exposure and gender, age group, years of experience and previous AI training were added to the reduced model one block at a time and tested with an HC3 Wald test. Common-method variance was assessed with Harman’s single-factor test across all 48 scale items. Statistical significance was set at two-sided *p* < 0.05, and at q < 0.05 for the exploratory family.

## 3. Results

Table 1 reports the exploratory group comparisons. Of the 30 tests, 11 survived Benjamini–Hochberg control of the false discovery rate at q < 0.05, and all but two of these involve the three AI-related variables. Age group, educational level, job title, nursing specialty and years of experience were not associated with any of the three scores (all q > 0.24), which is the same conclusion reached by the previous parametric analysis, and the numerical increase in readiness across age groups is not supported (q = 0.301).

City or region was associated with perceived ability to use AI (H = 13.07, q = 0.030) and with readiness (H = 15.45, q = 0.013), with small effect sizes (epsilon-squared 0.037 and 0.047). Men scored higher on readiness than women (q = 0.030, rank-biserial r = 0.22). The largest differences involve the AI variables. Most-used programme was associated with all three scores (all q < 0.001); Dunn comparisons with Benjamini–Hochberg adjustment show that every difference runs between ChatGPT 4.1 users and each of the other three groups (all q < 0.001 for readiness), with no reliable differences among Gemini 2.5, DeepSeek-R1 and the “others” group except DeepSeek-R1 versus others on readiness (q = 0.016). Nurses reporting previous AI training and nurses endorsing an AI course scored higher on all three measures, with very large rank effect sizes for readiness (r = 0.97 and r = 0.90). Those two comparisons rest on reference groups of 30 and 10 participants, respectively, and are the least stable results in the table. None of these differences can be read as an effect of a tool or of a course. Programme choice was not randomised, and “most-used programme” says nothing about how much, how or for what purpose a tool was used, so the between-programme contrasts describe self-selected groups that may differ in digital familiarity, access modality, education and region.

Table 2 reports the adjusted models with HC3 standard errors. In the reduced model, perceived ability to use AI remained associated with both outcomes after adjustment for all ten covariates. Each additional point was associated with 0.38 more productivity points (95% CI 0.28 to 0.49, *p* < 0.001; standardised beta 0.56) and 0.80 more readiness points (95% CI 0.45 to 1.14, *p* < 0.001; standardised beta 0.27). The positive associations specified in H1 and H2 remained statistically significant after covariate adjustment. The two AI-attitude variables carried large adjusted coefficients for readiness, with previous training at 33.9 points (95% CI 19.7 to 48.2) and endorsement of an AI course at 33.7 points (95% CI 12.8 to 54.6), but their confidence intervals are wide because the reference groups contain only 30 and 10 participants. These covariate associations are exploratory and should be interpreted cautiously given the marked imbalance between groups and the limited information collected about previous AI training. Reporting Gemini 2.5 rather than ChatGPT 4.1 as the most-used programme was associated with lower scores on both outcomes.

Model checking. Both models violate the constant-variance assumption (Breusch–Pagan LM = 90.9, df = 19, *p* < 0.001 for productivity; LM = 149.1, df = 19, *p* < 0.001 for readiness), which is why HC3 standard errors are used throughout rather than the conventional tests reported in the previous version. Multicollinearity was acceptable (maximum variance inflation factor 4.53 in the reduced model and 5.32 in the full model). No observation had a Cook’s distance above 1; 18 observations for productivity and 20 for readiness exceeded 4/n, and excluding them left the exposure coefficients essentially unchanged (0.39 and 0.67, respectively). The full model with all 30 predictor degrees of freedom is reported in Supplementary Table S2 and gives the same conclusions for the exposure (0.38, 95% CI 0.28 to 0.48 for productivity; 0.73, 95% CI 0.38 to 1.09 for readiness), while several of the sparse-category contrasts that appeared significant under the previous conventional tests, including education, job title and specialty, do not survive HC3 inference.

Sensitivity and moderation. Removing the two extremely imbalanced AI-attitude variables reduced the explained variance substantially, from 0.671 to 0.605 for productivity and from 0.751 to 0.505 for readiness, while the exposure coefficient rose (0.43, 95% CI 0.31 to 0.54, and 1.21, 95% CI 0.61 to 1.80). Much of the very high R^2^ in the readiness model therefore comes from two binary self-reports with very small reference groups, and the R^2^ values should not be read as evidence of a strong explanatory model. Cluster-robust standard errors using city as the clustering unit gave the same conclusion for the exposure (*p* < 0.001 for both outcomes), but with only five clusters this estimator is unreliable, and it is reported for completeness rather than as a solution to the clustering problem. In the moderation analyses, none of the product terms between the mean-centred exposure and gender, age group or years of experience reached significance for either outcome (all *p* > 0.09). One interaction, exposure by previous AI training for productivity, was nominally significant (F(1,229) = 4.20, *p* = 0.042) and added 1.0% to R^2^; given that it is one of eight tests and rests on 30 untrained participants, it is treated as a hypothesis for future work rather than as a finding. Harman’s single-factor test across the 48 items showed that the first unrotated factor accounted for 55.4% of the total item variance. This finding is compatible with common-method variance, but the percentage is not an estimate of common-method bias, and the test cannot establish whether or to what extent such bias affected the observed associations.

The 250 analysed participants were predominantly female (77.2%, *n* = 193). Participants were mainly from Riyadh (36.0%, *n* = 90) and Jouf (23.6%, *n* = 59), followed by Jeddah (16.4%, *n* = 41), Al Ahsa (14.8%, *n* = 37) and Arar (9.2%, *n* = 23). Age was recorded in categories: 33.6% (*n* = 84) were under 25, 28.4% (*n* = 71) were 25 to 35 and 38.0% (*n* = 95) were 36 or older. Most held a bachelor’s degree (67.6%, *n* = 169); 22.0% (*n* = 55) held a master’s degree, 8.8% (*n* = 22) a diploma and 1.6% (*n* = 4) a doctorate. Registered nurses formed the largest role group (32.4%, *n* = 81), followed by nurses in nursing education (22.8%, *n* = 57) and charge nurses (14.4%, *n* = 36); only four participants were infection control nurses. Intensive care (23.6%, *n* = 59) and primary health care (19.2%, *n* = 48) were the largest specialties, and only eight participants were oncology nurses. Most had five or more years of experience (61.2%, *n* = 153). ChatGPT 4.1 was the most-used application (66.0%, *n* = 165), followed by Gemini 2.5 (17.6%, *n* = 44), other or unspecified applications (8.4%, *n* = 21) and DeepSeek-R1 (8.0%, *n* = 20). The “others” category was a free-text field that was not coded further, so the tools it contains are unknown and it should not be read as a homogeneous group. Previous AI training was reported by 88.0% (*n* = 220), and 96.0% (*n* = 240) considered an AI course necessary; the small size of the two reference groups (*n* = 30 and *n* = 10) makes every estimate involving them imprecise (Table 3)

Scores on all three scales were high relative to their possible ranges, and all three were negatively skewed. Perceived ability to use AI averaged 27.95 (SD, 7.94) out of a possible 40, with a median of 30.0 (IQR 24.0 to 32.8) and observed scores covering the full 8 to 40 range. Self-reported productivity averaged 21.50 (SD, 5.42) out of 30, with a median of 23.0 (IQR 18.0 to 25.0) and a range of 6 to 30. Self-reported practice readiness averaged 109.92 (SD, 23.77) out of 136, with a median of 114.5 (IQR 102.0 to 129.0) and a range of 34 to 136. The readiness mean is 109.92; the value of 109.91 that appeared in the text of the previous version was a rounding error and every occurrence has been corrected. Reliability was high for all three scores (Table 4). The readiness alpha of 0.983 sits alongside a mean inter-item correlation of 0.637 and a one-factor solution accounting for 65.0% of item variance, which indicates item redundancy rather than validity, as set out in Section 2.3.4.

All three rank correlations were positive (Table 5). Perceived ability to use AI correlated with self-reported productivity (r_s = 0.638, 95% CI 0.529 to 0.727) and with self-reported practice readiness (r_s = 0.479, 95% CI 0.363 to 0.580), and productivity correlated with readiness (r_s = 0.642, 95% CI 0.548 to 0.721). H1 and H2 were therefore supported. These are correlations among three self-reports collected on a single occasion from the same respondent, and they carry no information about direction.

## 4. Discussion

This study asked whether nurses who rate themselves as more able to use AI tools also rate their own productivity and their own practice readiness more highly. They do. Nothing in the design allows a stronger statement than that, and the discussion below is written to stay within that limit. Three points frame everything that follows. First, the exposure is a self-appraisal of ability, not a property of any AI system, and the manuscript no longer uses the word “usability” for it. Second, participants named general-purpose generative tools (ChatGPT 4.1, Gemini 2.5, DeepSeek-R1) rather than clinical systems, and the questionnaire did not record whether those tools were used for clinical work at all, so the study cannot speak to AI integrated into clinical workflows, clinical decision support, documentation systems, triage, monitoring, or any contact with patient data. Third, both outcomes are self-reports collected from the same respondent on the same occasion using similarly worded agreement scales, so shared method effects and a general tendency to answer positively remain plausible explanations for the size of the correlations.

Participants rated their ability to use AI fairly highly, which is consistent with reports from Saudi Arabia and the wider region that health professionals and students with more exposure to AI applications hold more favourable views of them [6,24]. The comparison is limited, though, because those studies also measured perceptions rather than use, and because convenience recruitment at sites already engaged with the research team is likely to have attracted nurses with an existing interest in AI. The 88.0% reporting prior AI training and the 96.0% endorsing an AI course are almost certainly higher than the corresponding figures in the Saudi nursing workforce and should not be read as prevalence estimates.

Self-appraised productivity was also relatively high, in line with work reporting that perceived job performance in healthcare is associated with organisational efficiency and the availability of supportive technology [25,26]. It is worth stating explicitly that the productivity measure used here is an opinion. Nothing was recorded about documentation time, tasks completed, workload, time in direct care, errors or rework, so the widely cited mechanism by which AI is said to raise productivity, namely a reduced documentation burden and faster access to information [27,28], was not tested in this study and cannot be inferred from these data.

Self-reported practice readiness scores were high, but their interpretation is limited by the NPRS’s internal structure and its application to experienced practising nurses. The instrument was developed for students and new graduates in transition to practice, and its Arabic validation was carried out in Saudi nursing students [22,23]. In the present sample of practising nurses, 61.2% of whom had five or more years of experience, the five-factor structure did not reproduce: parallel analysis retained one factor, the first component accounted for 65.0% of item variance, and the mean inter-item correlation was 0.637. The most defensible reading is that among these participants the NPRS captured a single, positively toned self-appraisal of professional competence rather than readiness to enter practice, and that is how the score is labelled and interpreted throughout the revised manuscript. This is a substantive limitation on the transferability of the outcome, not a detail of scoring, and it is one reason why the readiness findings should be treated as preliminary. These findings do not establish the NPRS as a validated measure of professional readiness in experienced nurses or provide objective evidence of their clinical competence.

Perceived ability to use AI correlated with self-reported productivity, which is the pattern the Technology Acceptance Model would anticipate for perceived ease of use and perceived usefulness [29]. TAM is introduced here as one possible interpretive frame after the fact; its constructs were not measured, and the study does not test it, just as it does not test tasktechnology fit. Comparable survey work indicates that clinicians who find AI applications intuitive report using them more often and finding them more helpful [30]. Reverse explanations are at least as plausible in a cross-sectional design. Nurses who already feel efficient and competent may find any new tool easier to adopt, and a general disposition to answer positively would produce this correlation on its own.

Perceived ability to use AI also correlated with self-reported practice readiness, and the association persisted after adjustment. A defensible statement of the finding is that higher perceived ability to use AI was associated with a higher self-reported practice-readiness score, and that the cross-sectional design does not establish whether AI use improves technological literacy or clinical competence. Digital competence is increasingly discussed as part of contemporary professional preparedness [31,32,33], but that literature is about expectation and curriculum rather than demonstrated effect, and the present data cannot settle it.

Productivity and readiness were themselves strongly correlated. Two self-appraisals of one’s own work, answered minutes apart on similar scales, would be expected to correlate for reasons that have nothing to do with AI. Although the first unrotated factor accounted for 55.4% of the total item variance, Harman’s single-factor test provides only a crude diagnostic and cannot confirm or quantify common-method bias. The stronger reason for concern is that the predictor and both outcomes were collected simultaneously from the same respondents using similarly worded self-report scales. The correlation is reported because it was prespecified, but it should not be described as evidence that the two constructs reinforce one another, and the previous version’s description of perceived AI ability as a “strategic determinant of nursing performance” has been removed.

Perceived ability to use AI remained positively associated with both self-reported outcomes in the adjusted models and reported sensitivity analyses. Interpretation should focus on these exposure coefficients and their confidence intervals. The productivity coefficient changed little when the two severely imbalanced AI-related variables were excluded, increasing from 0.38 to 0.43. The readiness coefficient increased from 0.80 to 1.21, indicating that its magnitude was more sensitive to model specification, although the association remained positive. The overall R^2^ values were sensitive to these variables and should not be interpreted as evidence of strong explanatory performance.

Moving from Type III omnibus tests to coefficients with confidence intervals also changed which covariates appear important. Several contrasts that reached significance under the previous conventional tests, including educational level, job title and nursing specialty, did not survive HC3 inference, and none of them survived false-discovery-rate control in the bivariate analysis. The comparisons between users of different AI programmes are secondary, exploratory findings describing differences between self-selected groups. They cannot establish effects of platforms or differences in their effectiveness. Programme choice was not randomised, exposure intensity was not measured, and the “others” category was free text that was never coded, so no claim is made here about the relative effectiveness of any platform. The large coefficients attached to previous training and to endorsing an AI course are similarly ambiguous. These exploratory estimates rely on reference groups of only 30 participants without previous training and 10 participants who did not endorse an AI course. They should therefore be interpreted cautiously and should not be presented as robust independent predictors. Nurses who are more interested and more confident are more likely to seek out training, and nothing was recorded about the content, duration, provider or recency of that training, so the direction of that association cannot be resolved here.

One further point follows from what participants reported using. General-purpose generative models can help a nurse organise, summarise or rephrase knowledge that the nurse already holds and can verify, but they are not a dependable source of clinical guidance. They fabricate facts and references, return outdated content, carry bias from their training data, express uncertainty poorly and invite automation bias in users who are pressed for time. Entering patient information into a consumer tool raises separate privacy and governance problems, and accountability for a decision informed by such a tool remains with the clinician. The implication is that AI training for nurses cannot be reduced to teaching tool operation. It must cover critical appraisal of output, verification against authoritative sources, privacy and data handling, recognition of uncertainty, the limits of autonomous use, and the institutional governance under which any use is permitted. Because this study measured no patient outcome, no workload measure and no safety indicator, nothing here supports a claim that AI use improves the quality or safety of care.

### 4.1. Strengths of the Study

The study has three strengths. Recruitment spanned five cities or regions rather than a single facility. Arabic instruments with prior psychometric evaluation were used for all three constructs, and their properties were re-examined in the present sample rather than taken for granted. The analysis reported here uses robust inference, prespecified sensitivity analyses and false-discovery-rate control, and the raw data supporting it are available from the corresponding author under the conditions set out in the data availability statement.

### 4.2. Limitations

The limitations are more substantial, and they bound the conclusions. (1) Design. The survey is cross-sectional, and every association reported is compatible with the reverse ordering; nurses who feel more productive and more competent may simply find AI tools easier to approach. (2) Exposure definition. The questionnaire recorded only which application was used most often and two yes/no attitude items. Purpose, frequency, duration, clinical versus non-clinical context, institutional authorisation, integration into the electronic health record, supervision, use of patient data and access modality were all unmeasured, and no non-user option was offered. The study therefore cannot characterise AI use, and it treats a heterogeneous set of general-purpose tools as if it were a single exposure. (3) Outcome transferability. The NPRS was developed for students and new graduates and behaved as a single, highly redundant self-appraisal dimension in this sample of practising nurses. Its scores should therefore be interpreted as preliminary indicators of general professional self-appraisal. Future research should examine its factor structure and measurement invariance across experience and role groups before interpreting these scores as established measures of professional readiness in experienced nurses. (4) Measurement. Both outcomes are self-reports. No objective productivity indicator, patient outcome, workload measure or safety indicator was collected. (5) Common-method variance. The predictor and both outcomes were collected simultaneously from the same respondents using similarly worded self-report scales, creating a risk of shared method effects. Harman’s test cannot determine how much these effects contributed to the observed associations. (6) Sampling. Convenience sampling at sites already engaged with the research team, without a documented sampling frame, makes the sample unrepresentative of the Saudi nursing workforce and is the most likely reason for the very high AI-training and AI-endorsement proportions. (7) Clustering. Nurses are nested within hospitals, but hospital identifiers were not retained in the analysis file. City-level cluster-robust standard errors are reported as an approximation, and with five clusters that estimator is itself unreliable, so within-site dependence is acknowledged rather than resolved. (8) Sparse categories and residual confounding. Several categories were very small, and the two AI-attitude variables have reference groups of 30 and 10; staffing levels, workload, unit acuity, leadership support, digital infrastructure and organisational climate were not measured and may confound every association reported. (9) Subscale use. Only one MAIRS-MS subscale was administered, so the broader construct that the parent instrument was built to measure was not captured. (10) Multiplicity. Table 4 is exploratory; false-discovery-rate control was applied, but these comparisons still warrant replication.

### 4.3. Implications for Nursing Practice and Health Policy

These findings are hypothesis-generating, and the implications are framed accordingly. Where healthcare organisations are already deploying AI tools for nursing, the case for user-centred implementation that fits existing workflows and comes with reliable technical support rests on the wider implementation literature rather than on this study, which evaluated no system. What this study does suggest is that nurses who feel able to use these tools also feel better about their work, and that a formal training and governance route may be preferable to the informal, individually initiated use of consumer tools that the participants appear to describe. Any such training should cover critical appraisal, verification, privacy, accountability and bias alongside tool operation. Priorities for research are clearer than priorities for policy: studies that define which system is used, for which task and under which governance; longitudinal or intervention designs; and objective productivity, workload, safety and patient outcomes rather than self-reports alone. Until such evidence exists, the associations reported here are not a basis for procurement or curriculum decisions.

### 4.4. Significance of the Study

The contribution of this study is descriptive. It documents that among a convenience sample of nurses working in five Saudi cities, general-purpose generative AI tools are in widespread self-reported use, that ChatGPT 4.1 dominates, and that nurses who feel more able to use these tools rate their own productivity and their own professional readiness more highly. It also documents, using the raw data, two measurement problems that future Saudi work in this area will have to solve: the absence of any characterisation of what AI use consists of, and the behaviour of a student-derived readiness instrument when it is applied to experienced nurses. Making both problems explicit is, in our view, more useful to the field at this stage than a confident effect estimate would have been.

## 5. Conclusions

In a convenience sample of 250 nurses working in five Saudi cities, nurses who rated their own ability to use AI more highly also rated their own productivity and their own practice readiness more highly, and the two self-rated outcomes were correlated with each other. These associations persisted after adjustment for ten sociodemographic, professional and AI-related covariates using robust standard errors. Because the design is cross-sectional, the exposure was a self-appraisal rather than a property of any AI system, the tools involved were general-purpose generative models of unknown clinical use, both outcomes were self-reported, and the readiness instrument did not reproduce its published structure in this sample, the findings should be read as correlations among self-reports. They do not show that AI use raises nursing productivity or professional competence, and they do not support conclusions about the quality or safety of patient care. The exploratory comparisons between self-selected programme-user groups provide no basis for recommending one AI platform over another. Work that defines the technology, the task and the governance context, and that measures objective outcomes over time, is needed before practice or policy recommendations can be drawn.

## Figures and Tables

**Table 1:** Exploratory comparisons of the three scale scores across participant characteristics (*n* = 250).

Participant Characteristic	Perceived Ability to Use AI, Median (IQR)	Test, *p*, q, Effect Size	Self-Reported Nursing Productivity, Median (IQR)	Test, *p*, q, Effect Size	Self-Reported Practice Readiness, Median (IQR)	Test, *p*, q, Effect Size
Gender		U = 6041, *p* = 0.258, q = 0.369, r = 0.10		U = 6112, *p* = 0.200, q = 0.333, r = 0.11		U = 6726, *p* = 0.011, q = 0.030, r = 0.22
Male (*n* = 57)	31.0 (24–35)		24.0 (19–26)		118.0 (104–133)	
Female (*n* = 193)	30.0 (24–32)		23.0 (18–25)		113.0 (102–128)	
City or region		H = 13.07, *p* = 0.011, q = 0.030, ε^2^ = 0.037		H = 8.33, *p* = 0.080, q = 0.185, ε^2^ = 0.018		H = 15.45, *p* = 0.004, q = 0.013, ε^2^ = 0.047
Arar (*n* = 23)	27.0 (24–32)		24.0 (18–24)		131.0 (102–136)	
Al Ahsa (*n* = 37)	30.0 (25–32)		19.0 (17–24)		102.0 (82–117)	
Jeddah (*n* = 41)	32.0 (31–35)		24.0 (18–26)		115.0 (106–131)	
Jouf (*n* = 59)	31.0 (24–34)		23.0 (18–24)		113.0 (102–129)	
Riyadh (*n* = 90)	27.0 (21–32)		22.5 (18–25)		115.0 (102–128)	
Age group		H = 0.94, *p* = 0.625, q = 0.694, ε^2^ = 0.000		H = 0.69, *p* = 0.709, q = 0.759, ε^2^ = 0.000		H = 3.54, *p* = 0.170, q = 0.301, ε^2^ = 0.006
<25 years (*n* = 84)	30.0 (22–34)		22.5 (18–24)		110.5 (98–128)	
25–35 years (*n* = 71)	29.0 (23–32)		23.0 (18–25)		115.0 (100–131)	
≥36 years (*n* = 95)	31.0 (24–32)		23.0 (18–25)		115.0 (102–131)	
Educational level		H = 2.78, *p* = 0.427, q = 0.512, ε^2^ = 0.000		H = 5.94, *p* = 0.114, q = 0.245, ε^2^ = 0.012		H = 5.64, *p* = 0.130, q = 0.261, ε^2^ = 0.011
Diploma (*n* = 22)	28.0 (22–32)		21.0 (17–25)		102.0 (92–121)	
Bachelor (*n* = 169)	30.0 (24–33)		24.0 (18–25)		115.0 (102–130)	
Master (*n* = 55)	31.0 (24–32)		21.0 (18–24)		115.0 (106–126)	
Doctorate (*n* = 4)	36.0 (30–37)		23.5 (15–30)		109.5 (92–117)	
Job title		H = 7.75, *p* = 0.257, q = 0.369, ε^2^ = 0.007		H = 3.56, *p* = 0.736, q = 0.761, ε^2^ = 0.000		H = 1.73, *p* = 0.942, q = 0.942, ε^2^ = 0.000
Assistant Nurse (*n* = 20)	23.0 (16–31)		21.5 (18–24)		111.0 (100–129)	
Registered Nurse (*n* = 81)	30.0 (22–32)		22.0 (18–24)		115.0 (96–128)	
Charge Nurse (*n* = 36)	32.0 (26–32)		24.0 (18–25)		115.0 (96–132)	
Head Nurse (*n* = 31)	31.0 (26–37)		24.0 (18–26)		117.0 (108–132)	
Nursing Education (*n* = 57)	32.0 (24–33)		23.0 (18–24)		113.0 (102–126)	
Quality Nurse (*n* = 21)	28.0 (24–32)		22.0 (18–24)		107.0 (102–124)	
Infection Control Nurse (*n* = 4)	21.5 (15–30)		24.0 (23–24)		122.5 (92–133)	
Nursing specialty		H = 8.44, *p* = 0.296, q = 0.403, ε^2^ = 0.006		H = 7.24, *p* = 0.404, q = 0.506, ε^2^ = 0.001		H = 9.35, *p* = 0.229, q = 0.361, ε^2^ = 0.010
Acute (*n* = 23)	24.0 (12–32)		18.0 (14–24)		109.0 (94–136)	
ER (*n* = 31)	28.0 (26–32)		23.0 (18–25)		113.0 (96–126)	
ICU (*n* = 59)	31.0 (27–33)		24.0 (19–24)		115.0 (106–128)	
Primary healthcare (*n* = 48)	28.0 (22–32)		21.0 (18–24)		117.0 (102–124)	
Surgical (*n* = 30)	27.0 (24–32)		21.5 (18–24)		107.0 (96–126)	
Midwifery (*n* = 15)	32.0 (25–32)		21.0 (20–24)		101.0 (84–126)	
Oncology (*n* = 8)	33.0 (30–34)		23.0 (18–24)		116.0 (106–123)	
General (*n* = 36)	32.0 (24–35)		24.5 (19–27)		115.5 (108–132)	
Years of experience		U = 7842, *p* = 0.448, q = 0.517, r = 0.06		U = 6898, *p* = 0.346, q = 0.451, r = 0.07		U = 6639, *p* = 0.160, q = 0.301, r = 0.11
<5 years (*n* = 97)	30.0 (22–35)		22.0 (18–24)		113.0 (101–128)	
≥5 years (*n* = 153)	30.0 (24–32)		23.0 (18–25)		115.0 (102–131)	
Most-used AI programme		H = 38.49, *p* = <0.001, q = <0.001, ε^2^ = 0.144		H = 67.98, *p* = <0.001, q = <0.001, ε^2^ = 0.264		H = 105.25, *p* = <0.001, q = <0.001, ε^2^ = 0.416
ChatGPT 4.1 (*n* = 165)	32.0 (27–35)		24.0 (21–26)		122.0 (113–131)	
Gemini 2.5 (*n* = 44)	24.0 (24–32)		18.0 (18–23)		102.0 (101–102)	
DeepSeek-R1 (*n* = 20)	23.0 (19–26)		17.0 (15–18)		87.0 (82–92)	
Others (*n* = 21)	22.0 (11–30)		18.0 (11–24)		68.0 (53–136)	
Previous AI training		U = 5112, *p* = <0.001, q = <0.001, r = 0.55		U = 5828, *p* = <0.001, q = <0.001, r = 0.77		U = 6502, *p* = <0.001, q = <0.001, r = 0.97
Yes (*n* = 220)	31.0 (24–33)		24.0 (19–25)		116.0 (105–131)	
No (*n* = 30)	21.0 (11–28)		15.0 (11–18)		68.0 (52–82)	
Perceived need for an AI course		U = 1702, *p* = 0.025, q = 0.062, r = 0.42		U = 2014, *p* = <0.001, q = <0.001, r = 0.68		U = 2284, *p* = <0.001, q = <0.001, r = 0.90
Yes (*n* = 240)	31.0 (24–33)		23.0 (18–25)		115.0 (102–129)	
No (*n* = 10)	17.0 (8–31)		9.5 (6–15)		42.5 (34–61)	

Note: U, Mann–Whitney U; H, Kruskal–Wallis H; r, rank-biserial correlation; ε^2^, epsilon-squared; IQR, interquartile range. q values are Benjamini–Hochberg false-discovery-rate adjusted across the full family of 30 tests in this table. All comparisons in this table are exploratory.

**Table 2:** Multivariable linear models for self-reported nursing productivity and self-reported practice readiness, with HC3 heteroscedasticity-consistent standard errors (*n* = 250).

Predictor	Self-Reported Nursing Productivity: B (95% CI)	*P*	Self-Reported Practice Readiness: B (95% CI)	*p*
Intercept	3.37 (−1.04 to 7.78)	0.134	32.83 (9.17 to 56.50)	0.007
Perceived ability to use AI (per 1 point)	0.38 (0.28 to 0.49)	<0.001	0.80 (0.45 to 1.14)	<0.001
Male (ref. female)	−0.34 (−1.57 to 0.89)	0.588	4.00 (−0.48 to 8.47)	0.080
Al Ahsa (ref. Arar)	−1.04 (−3.19 to 1.10)	0.339	−8.60 (−17.32 to 0.11)	0.053
Jeddah (ref. Arar)	−0.45 (−2.51 to 1.60)	0.665	−6.57 (−14.44 to 1.30)	0.101
Jouf (ref. Arar)	0.73 (−1.34 to 2.80)	0.487	−11.50 (−19.39 to −3.61)	0.004
Riyadh (ref. Arar)	−0.08 (−1.91 to 1.75)	0.931	−8.12 (−16.32 to 0.08)	0.052
Age < 25 years (ref. 25–35)	0.07 (−1.68 to 1.82)	0.934	5.19 (−0.30 to 10.68)	0.064
Age ≥ 36 years (ref. 25–35)	−1.39 (−2.57 to −0.22)	0.021	−1.87 (−6.61 to 2.86)	0.437
Diploma (ref. bachelor)	−0.88 (−2.43 to 0.67)	0.263	−5.21 (−11.76 to 1.34)	0.119
Postgraduate (ref. bachelor)	−2.98 (−4.12 to −1.84)	<0.001	−3.06 (−8.14 to 2.03)	0.237
Educator, quality or IPC role (ref. bedside)	0.60 (−0.52 to 1.72)	0.291	−2.55 (−6.99 to 1.90)	0.261
Supervisory role (ref. bedside)	−0.55 (−1.69 to 0.59)	0.342	−1.88 (−7.14 to 3.38)	0.482
Primary health care or general (ref. critical/acute)	0.79 (−0.36 to 1.93)	0.176	3.48 (−0.84 to 7.80)	0.113
Surgical, oncology or midwifery (ref. critical/acute)	0.10 (−1.13 to 1.33)	0.876	−0.09 (−4.73 to 4.55)	0.969
≥5 years experience (ref. < 5)	2.87 (0.77 to 4.96)	0.008	6.76 (0.82 to 12.69)	0.026
Gemini 2.5 most used (ref. ChatGPT 4.1)	−2.67 (−3.82 to −1.52)	<0.001	−16.72 (−20.75 to −12.68)	<0.001
Other or unspecified (ref. ChatGPT 4.1)	−2.13 (−4.48 to 0.22)	0.075	−7.50 (−19.44 to 4.44)	0.217
Previous AI training, yes (ref. no)	2.50 (−0.08 to 5.08)	0.058	33.94 (19.67 to 48.20)	<0.001
AI course seen as necessary, yes (ref. no)	5.70 (2.23 to 9.17)	0.001	33.67 (12.77 to 54.58)	0.002
Model R^2^ (adjusted R^2^)	0.671 (0.643)		0.751 (0.731)	

Note: B, unstandardized regression coefficient; CI, confidence interval. Standard errors, *p* values and confidence intervals are HC3 heteroscedasticity-consistent. Reference categories are shown in parentheses. Perceived ability to use AI is a continuous covariate; all other predictors are indicator variables. Sparse categories were combined before analysis as described in Section 2.5; the uncombined 30-degree-of-freedom model, together with the Type III omnibus tests and partial eta-squared values, is in Supplementary Table S2. IPC, infection prevention and control.

**Table 3:** Personal and professional characteristics of the participating nurses (*n* = 250).

Personal Data	Frequency	Percentage
Gender		
Male	57	22.8
Female	193	77.2
City		
Arar	23	9.2
Al Ahsa	37	14.8
Jeddah	41	16.4
Jouf	59	23.6
Riyadh	90	36.0
Age		
<25 years	84	33.6
25–35 years	71	28.4
≥36	95	38.0
Educational Level		
Bachelor’s degree	169	67.6
Diploma of Nursing	22	8.8
Doctorate degree	4	1.6
Master’s degree	55	22.0
Job title		
Assistant Nurse	20	8.0
Charge Nurse	36	14.4
Head Nurse	31	12.4
Infection Control Nurse	4	1.6
Nursing Education	57	22.8
Quality Nurse	21	8.4
Registered Nurse	81	32.4
Nursing Specialty		
Acute nurse	23	9.2
ER nurse	31	12.4
ICU nurse	59	23.6
Primary healthcare nurse	48	19.2
Surgical nurse	30	12.0
Midwifery nurse	15	6.0
Oncology nurse	8	3.2
General nurses	36	14.4
Years of Experience		
Less than 5 years	97	38.8
≥5 years	153	61.2
What AI programme is used the most?		
ChatGPT 4.1	165	66.0
Gemini 2.5	44	17.6
DeepSeek-R1	20	8.0
Others	21	8.4
Did you attend training courses about AI usage in nursing previously?		
Yes	220	88.0
No	30	12.0
Do you think it is necessary to incorporate a course on the use of artificial intelligence in nursing to practice?		
Yes	240	96.0
No	10	4.0

**Table 4:** Descriptive statistics, reliability and convergent validity for the three scale scores (*n* = 250).

Study Variable	Mean (SD)	Median (IQR)	Cronbach α	McDonald ω	CR	AVE
Perceived ability to use AI (range 8–40)	27.95 (7.94)	30.0 (24.0–32.8)	0.952	0.952	0.952	0.714
Self-reported nursing productivity (range 6–30)	21.50 (5.42)	23.0 (18.0–25.0)	0.869	0.869	0.869	0.529
Self-reported practice readiness (range 34–136)	109.92 (23.77)	114.5 (102.0–129.0)	0.983	0.983	0.983	0.636

Note: SD, standard deviation; IQR, interquartile range; CR, composite reliability; AVE, average variance extracted. Minimum and maximum are observed values.

**Table 5:** Spearman rank correlations with percentile bootstrap confidence intervals (*n* = 250, 5000 resamples).

Pair of Variables	r_s	95% CI (Bootstrap)	*p*
Perceived ability to use AI and self-reported productivity	0.638	0.529 to 0.727	<0.001
Perceived ability to use AI and self-reported practice readiness	0.479	0.363 to 0.580	<0.001
Self-reported productivity and self-reported practice readiness	0.642	0.550 to 0.721	<0.001

Note: r_s, Spearman rank correlation coefficient; CI, confidence interval.

## Data Availability

The participant-level data supporting the findings of this study are not publicly available because the informed consent and ethical approvals did not include unrestricted public data sharing. Although direct identifiers were not included in the analytical dataset, combinations of demographic and professional variables could create a risk of indirect participant identification, particularly within small subgroups. A suitably de-identified dataset may be made available by the corresponding author upon reasonable request, subject to applicable ethical and institutional requirements and the completion of an appropriate data-use agreement.

## Supplementary Materials

The following supporting information can be downloaded at: https://www.mdpi.com/article/10.3390/healthcare14183065/s1, Table S1: the eight items of the perceived ability to use AI measure, in Arabic and English. Table S2: the full 30-degree-of-freedom adjusted models with HC3 coefficients and the Type III omnibus tests with partial eta-squared (panel A and B). File S3: completed STROBE checklist for cross-sectional studies.

## Abbreviations

AI, artificial intelligence; AVE, average variance extracted; CR, composite reliability; ER, emergency room; ICU, intensive care unit; IRB, Institutional Review Board; MAIRS-MS, Medical Artificial Intelligence Readiness Scale for Medical Students; NPRS, Nursing Practice Readiness Scale; SD, standard deviation; SPSS, Statistical Package for the Social Sciences; STROBE, Strengthening the Reporting of Observational Studies in Epidemiology; TTF, task–technology fit.
