# Supplementary material for: The Association Between Perceived Ability to Use Artificial Intelligence, Self-Reported Nursing Productivity, and Self-Reported Practice Readiness Among Nurses in Saudi Arabia: A Multicentre Cross-Sectional Study
Source: Healthcare (Basel). 2026 Sep 17;14(18):3065. doi: 10.3390/healthcare14183065 (PMC13606050; doi:10.3390/healthcare14183065)
Supplement: Supplementary file 1 [file healthcare-14-03065-s001.zip › healthcare-4520402-supplementary.pdf]

Supplementary Materials

Table S1. Items of the perceived ability to use AI measure

Source: the eight-item "utilization of AI in nursing jobs" subscale of the Arabic instrument validated by Alenezi, Alshammari and Ibrahim (2024), adapted from the Medical Artificial Intelligence Readiness Scale for Medical Students (MAIRS-MS; Karaca, Calışkan and Demir, 2021). Responses are on a five-point Likert scale from 1 (strongly disagree) to 5 (strongly agree). Total score range 8 to 40. Higher scores indicate greater perceived ability to use AI in nursing work. In the present sample, Cronbach's alpha = 0.952, McDonald's omega = 0.952, composite reliability = 0.952, average variance extracted = 0.714, one-factor loadings 0.82 to 0.89, SRMR = 0.029.

Item	Arabic (as administered)	English translation
AI.Use1	أستطيع استخدام المعلومات القائمة على الذكاء الاصطناعي بالتكامل مع معرفتي المهنية.	I can use AI-based information in combination with my professional knowledge.
AI.Use2	أستطيع استخدام تقنيات الذكاء الاصطناعي بفعالية وكفاءة في تقديم الرعاية الصحية.	I can use AI technologies effectively and efficiently in healthcare delivery.
AI.Use3	أستطيع استخدام تطبيقات الذكاء الاصطناعي بما يتوافق مع الغرض من استخدامها.	I can use artificial intelligence applications in accordance with its purpose.
AI.Use4	أستطيع الوصول إلى المعرفة، وتقييمها، واستخدامها، ومشاركتها، وإنشاء معرفة جديدة باستخدام تقنيات المعلومات والاتصالات.	I can access, evaluate, use, share and create new knowledge using information and communication technologies.
AI.Use5	أستطيع توضيح المشكلة التي تقدم تطبيقات الذكاء الاصطناعي في الرعاية الصحية حلولًا لها.	I can explain how AI applications in healthcare offer a solution to which problem.
AI.Use6	أرى أن استخدام الذكاء الاصطناعي له قيمة في أغراض التعليم والخدمات والبحث العلمي.	I find it valuable to use AI for education, service and research purposes.
AI.Use7	أستطيع شرح تطبيقات الذكاء الاصطناعي المستخدمة في خدمات الرعاية الصحية للمريض.	I can explain the AI applications used in healthcare services to the patient.
AI.Use8	أستطيع اختيار تطبيق الذكاء الاصطناعي المناسب للمشكلة التي تواجهني في مجال الرعاية الصحية.	I can choose the proper AI application for the problem encountered in healthcare.

Note: the item labels in the left column match the variable names in the analysis dataset, so that each administered item can be traced to the data.

Table S2. Full adjusted models (30 predictor degrees of freedom)

These are the uncombined models, retaining every original category including doctorate holders (n = 4), infection control nurses (n = 4) and oncology nurses (n = 8). The reduced models reported as Table 5 in the manuscript, in which these sparse categories are combined on clinical grounds before estimation, are the primary analysis. Both are shown so that readers can see that the exposure estimate is unaffected by the choice, while several sparse-category contrasts are not stable.

Panel A. Unstandardized coefficients with HC3 heteroscedasticity-consistent 95% confidence intervals

Predictor	Productivity B (95% CI)	p	Practice readiness B (95% CI)	p
Intercept	2.33 (-2.63 to 7.30)	0.356	33.86 (3.59 to 64.13)	0.029
Perceived ability to use AI (per 1 point)	0.38 (0.28 to 0.48)	<0.001	0.73 (0.38 to 1.09)	<0.001
Male (ref. female)	-0.31 (-1.76 to 1.15)	0.677	6.11 (0.16 to 12.06)	0.044
Eastern Region (ref. Arar)	-1.46 (-4.16 to 1.25)	0.289	-11.72 (-21.76 to -1.68)	0.022
Jeddah (ref. Arar)	-1.12 (-3.37 to 1.13)	0.328	-8.17 (-17.51 to 1.16)	0.086
Jouf (ref. Arar)	0.12 (-2.25 to 2.50)	0.918	-15.51 (-25.83 to -5.19)	0.003
Riyadh (ref. Arar)	-0.63 (-2.88 to 1.61)	0.580	-11.18 (-21.40 to -0.96)	0.032
Age <25 years (ref. 25-35)	0.80 (-1.15 to 2.75)	0.422	6.74 (-0.04 to 13.52)	0.051
Age ≥36 years (ref. 25-35)	-1.36 (-2.79 to 0.07)	0.062	-2.54 (-7.43 to 2.34)	0.306
Diploma (ref. bachelor)	-0.97 (-2.74 to 0.81)	0.286	-7.88 (-14.56 to -1.20)	0.021
Doctorate (ref. bachelor), n = 4	-1.48 (-7.12 to 4.16)	0.606	-7.29 (-17.66 to 3.08)	0.167
Master (ref. bachelor)	-2.82 (-4.04 to -1.61)	<0.001	-1.07 (-6.69 to 4.56)	0.708
Charge nurse (ref. assistant nurse)	-1.93 (-4.57 to 0.70)	0.150	3.03 (-6.33 to 12.39)	0.524
Head nurse (ref. assistant nurse)	-2.55 (-5.30 to 0.21)	0.070	-9.42 (-19.70 to 0.86)	0.072
Infection control nurse (ref. assistant nurse), n = 4	0.31 (-7.41 to 8.04)	0.936	-11.91 (-35.86 to 12.03)	0.328
Nursing education (ref. assistant nurse)	-1.01 (-3.66 to 1.64)	0.452	-3.29 (-12.29 to 5.72)	0.473
Quality nurse (ref. assistant nurse)	-1.72 (-4.01 to 0.56)	0.139	-7.30 (-16.60 to 1.99)	0.123
Registered nurse (ref. assistant nurse)	-1.86 (-4.21 to 0.50)	0.121	-0.74 (-8.92 to 7.43)	0.858
Emergency (ref. acute)	2.36 (-0.27 to 4.99)	0.079	3.49 (-7.10 to 14.08)	0.517
General (ref. acute)	2.87 (0.30 to 5.44)	0.029	4.20 (-5.66 to 14.06)	0.402
Intensive care (ref. acute)	2.09 (-0.51 to 4.69)	0.114	2.01 (-8.38 to 12.39)	0.704
Midwifery (ref. acute)	2.76 (-0.17 to 5.69)	0.065	4.98 (-5.46 to 15.41)	0.348
Oncology (ref. acute), n = 8	0.21 (-2.86 to 3.28)	0.892	-3.99 (-17.94 to 9.95)	0.573
Primary health care (ref. acute)	2.36 (-0.08 to 4.80)	0.058	7.40 (-1.89 to 16.69)	0.118
Surgical (ref. acute)	2.20 (-0.96 to 5.36)	0.171	0.65 (-10.09 to 11.39)	0.906
≥5 years experience (ref. <5)	3.51 (1.35 to 5.68)	0.002	6.84 (0.06 to 13.63)	0.048
DeepSeek most used (ref. ChatGPT)	-2.74 (-5.18 to -0.29)	0.028	-10.62 (-22.27 to 1.03)	0.074
Gemini most used (ref. ChatGPT)	-2.80 (-4.09 to -1.52)	<0.001	-18.05 (-22.79 to -13.32)	<0.001
Others most used (ref. ChatGPT)	-0.59 (-3.72 to 2.55)	0.712	-5.17 (-23.33 to 13.00)	0.576
Previous AI training, yes (ref. no), reference n = 30	3.22 (0.77 to 5.67)	0.010	35.18 (20.55 to 49.82)	<0.001
AI course seen as necessary, yes (ref. no), reference n = 10	5.72 (2.43 to 9.01)	<0.001	34.75 (13.70 to 55.79)	0.001
Model R^2 (adjusted R^2)	0.699 (0.658)		0.775 (0.744)	

Note: B, unstandardized coefficient; CI, confidence interval. Standard errors, p values and confidence intervals are HC3 heteroscedasticity-consistent, because both models violate the constant-variance assumption (Breusch-Pagan LM = 113.8, df = 30, $p < 0.001$ for productivity; LM = 161.5, df = 30, $p < 0.001$ for practice readiness). Maximum variance inflation factor 5.32. No observation exceeded a Cook's distance of 1. Reference categories are shown in parentheses; the number of participants is given for categories with fewer than ten members, whose estimates should not be interpreted individually.

Panel B. Type III omnibus tests and partial eta-squared

Retained for completeness and for comparison with the previous version of the manuscript. These tests indicate that a term contributes to the model, but they do not show the direction or the magnitude of its association, which is why Panel A and Table 5 are the basis for interpretation. Type III sums of squares test each term after every other term, which is appropriate here because the design is non-orthogonal with markedly unequal cell sizes. Note that these conventional tests do not correct for the heteroscedasticity documented above; several terms significant here are not significant under HC3 inference.

**Source**	**Productivity Type III SS**	**df**	**F**	**p**	**ηp^2**	**Readiness Type III SS**	**df**	**F**	**p**	**ηp^2**
Corrected model	5105.81	30	16.937	<0.001	0.699	108944.12	30	25.078	<0.001	0.775
Gender	2.44	1	0.243	0.622	0.001	961.47	1	6.640	0.011	0.029
City or region	42.88	4	1.067	0.374	0.019	2256.03	4	3.895	0.004	0.066
Age group	72.04	2	3.585	0.029	0.032	968.49	2	3.344	0.037	0.030
Educational level	210.75	3	6.991	<0.001	0.087	944.25	3	2.174	0.092	0.029
Job title	100.11	6	1.660	0.132	0.044	2800.29	6	3.223	0.005	0.081
Nursing specialty	125.43	7	1.783	0.092	0.054	1214.51	7	1.198	0.305	0.037
Years of experience	184.83	1	18.393	<0.001	0.077	700.49	1	4.837	0.029	0.022
Most-used AI program	272.90	3	9.052	<0.001	0.110	9475.22	3	21.811	<0.001	0.230
Previous AI training	111.87	1	11.133	<0.001	0.048	13371.08	1	92.336	<0.001	0.297
Perceived necessity of AI course	227.55	1	22.644	<0.001	0.094	8395.75	1	57.978	<0.001	0.209
Perceived ability to use AI	1531.62	1	152.417	<0.001	0.410	5736.87	1	39.617	<0.001	0.153
Error	2200.69	219				31713.12	219			

Note: SS, sum of squares; ηp^2, partial eta-squared. Residual degrees of freedom = 219 for both models. These values reproduce the IBM SPSS Statistics 27 GLM output exactly, confirming that the design matrix used in File S4 matches the SPSS parameterisation.

File S3. STROBE checklist for cross-sectional studies

Completed against the revised manuscript. The section numbers refer to the revised, highlighted version. Three items depend on recruitment figures that the study team must supply and are marked accordingly.

#	STROBE recommendation	Reported in	Status
1a	Indicate the design with a commonly used term in the title or abstract	Title; Abstract, Methods	Yes
1b	Give a balanced summary of what was done and what was found in the abstract	Abstract	Yes; the abstract now states the limits on interpretation
2	Explain the scientific background and rationale	Section 1, paragraphs 1 to 4	Yes
3	State specific objectives, including any prespecified hypotheses	Sections 1.1 and 1.2 (H1 to H3, RQ1 to RQ3)	Yes; hypotheses added at revision
4	Present key elements of study design early in the paper	Section 2.1	Yes
5	Describe the setting, locations, and relevant dates	Section 2.1	yes
6a	Give the eligibility criteria, and the sources and methods of selection	Section 2.2	Yes; convenience sampling stated
7	Clearly define all outcomes, exposures, predictors, confounders, effect modifiers	Sections 1.1, 2.3.1 to 2.3.4	Yes; exposure relabelled and its limits stated
8	For each variable, give sources of data and details of methods of assessment	Sections 2.3.1 to 2.3.4	Yes; unmeasured AI-use dimensions listed explicitly
9	Describe any efforts to address potential sources of bias	Sections 2.1, 2.5, 4.2	Yes; common-method variance tested, HC3 and FDR applied
10	Explain how the study size was arrived at	Section 2.2	Yes
11	Explain how quantitative variables were handled in the analyses	Sections 2.3.1, 2.5	Yes; age and experience were collected categorically
12a	Describe all statistical methods, including those used to control for confounding	Section 2.5	Yes
12b	Describe any methods used to examine subgroups and interactions	Section 2.5; Results, moderation	Yes; added at revision
12c	Explain how missing data were addressed	Sections 2.1, 2.2	Yes; no missing values in the analysed dataset
12d	Describe analytical methods taking account of sampling strategy	Section 2.5	Partly; city-level cluster-robust SE, hospital ID unavailable
12e	Describe any sensitivity analyses	Section 2.5; Results	Yes; four prespecified sensitivity analyses
13a	Report numbers of individuals at each stage of the study	Section 3	yes
13b	Give reasons for non-participation at each stage	Section 3	yes
13c	Consider use of a flow diagram	Section 3	yes
14a	Give characteristics of study participants and information on exposures	Section 3; Table 1	Yes
14b	Indicate the number of participants with missing data for each variable	Sections 2.1, 3	Yes; none
15	Report numbers of outcome events or summary measures	Section 3; Tables 2 to 4	Yes; median and IQR added at revision
16a	Give unadjusted and confounder-adjusted estimates with their precision	Tables 3 to 5; Table S2	Yes; coefficients with 95% CI added at revision
16b	Report category boundaries when continuous variables were categorized	Section 2.3.1; Table 1	Yes
17	Report other analyses done	Section 2.5; Results	Yes; sensitivity, moderation, Harman, Dunn, FDR
18	Summarise key results with reference to study objectives	Section 4, opening; Section 5	Yes
19	Discuss limitations, direction and magnitude of any potential bias	Section 4.2 (ten numbered limitations)	Yes; expanded at revision
20	Give a cautious overall interpretation of results	Sections 4, 4.3, 5	Yes; all causal language removed
21	Discuss the generalisability of the study results	Sections 4.2, 4.3	Yes
22	Give the source of funding and the role of the funders	Funding statement	Yes; no specific funding

Note: this table records where each STROBE item is addressed. The journal may also require the official STROBE checklist form with page and line numbers, which should be completed from the final typeset line numbering.
